# Entropy generation analysis for mixed convection flow of nanofluid due to vertical plate with chemical reaction effects

**DOI:** 10.1038/s41598-023-39693-3

**Published:** 2023-08-16

**Authors:** T. N. Abdelhameed

**Affiliations:** 1 Basic Engineering Sciences Department, College of Engineering, Majmaah University, 11952 Majmaah, Saudi Arabia; 2 Mathematics Department, Faculty of Science, Beni-Suef University, Beni-Suef, 62514 Egypt

**Keywords:** Applied mathematics, Engineering, Physics

## Abstract

The analysis have been presented to observe the optimized flow of Casson nanofluid conveying the applications of external heat generation and mixed convection features. The problem is further influenced by chemical reactive species with order one. The significant of Bejan number is evaluated. A vertically moving with convective heat phenomenon endorsed the flow. The modeled problem is reflected in terms PDE’s which are further simplifies with dimensionless form. The analytical outcomes have been established with implementation of Laplace technique. The graphical impact conveying the different parameters is assessed. The insight of skin friction and Nusselt number is observed via various curves. It is observed that entropy generation enhanced due to porosity parameter and magnetic number. With increasing Casson fluid parameter, the entropy generation decrease. Moreover, the Bejan number decreases for chemical reaction constant.

## Introduction

The nanomaterials report extra exclusive thermal mechanism in contrasting to other base fluids. With low size, the nanoparticles prescribed more thermal performances and enhancing impact. The idea of nanofluid is based on experimental verifications and extra thermal results. The nanofluids are decomposed materials containing the tiny particles and base fluids. With unique thermal performances, such dispersed nanomaterials show excellent potentials in the heat exchangers, electronic cooling, solar systems, control systems etc. Many researchers devoted special attention on exploring the nanofluids properties. Khan et al.^1^ discussed the exclusive features of nanofluids which report enhancement in the power systems and elctrinc charge systems. Ali et al.^2^ reported iron zinc nanoparticles via spectroscopic approach. Qayyum et al.^3^ examined the blood fluid properties for suspension of MWCNTs via stretching disk. Khan et al.^4^ exclusively determined the Maxwell nanofluid thermal effects for boosting the heating capacitance. The reaction consequences for wavy surface with nanofluid flow was identified by Javed et al.^5^. Abbasi et al.^6^ studied the Carreau nanofluid following the electro‐osmotic phenomenon. Vaidya et al.^7^ reported the MHD outcomes for variable thermal flow of Phan–Thien–Tanner nanofluid. In another continuation, Vaidya et al.^8^ investigated the Rabinowitsch nanofluid flow and suggested the applicable impact on hydraulic systems. Maatoug et al.^9^ explained the lubricated surface flow of nanofluid. Khaled et al.^10^ examined the heating management of silver nanoparticles under the fluctuation of different layers.

The dynamic of heat transfer phenomenon is quite necessary in various thermal systems, engineering processes and industrial framework. The thermal phenomenon exclusively altered when a fluctuation in the heat transfer is observed. On this end, the optimized framework of heat transfer pattern is important to control the loss of heat transfer. The thermal optimization is important to meet the excellent thermal framework. The sustainability of various electronics devices and engineering systems reflects the attention of optimized phenomenon for attaining the desirable thermal efficiencies. In order to enhance and observe the thermal impact of designed systems and extrusion phenomenon is justified by laws of thermodynamics. According the first hypothesis, the no energy loss about the transmission of thermal energy. However, the shortcoming of this concept is associated to the explanation of energy generation. On this end, the second thermodynamics law claim about the goal of energy and controls the energy loss. This concept retains enchantment in thermal mechanism of systems. Exclusive applications of energy generation are observed in thermal systems, heat transfer devices, nuclear engineering, chemical processes etc. The basic understand of entropy generation (EG) was predicted by Bejan^11^. Abdelhameed^12^ addressed the MHD flow via accelerating plate conveying the entropy generation (EG) effects. Turkyilmazoglu^13^ predicted the onset of optimized framework with enrollment of velocity slip. Khan et al.^14^ pronounced insight of EG phenomenon regarding the Casson rheological material with reactive species. Kumar et al.^15^ intended the channel flow with entropy production due to hybrid nanofluid flow. The effects of steric for curved surface nanochannel flow justifying the EG applications are contributed by Liu et al.^16^. Shah et al.^17^ reported the EG applications for claiming the control of heating phenomenon due to curved moving surface. Iftikhar et al. ^18^ examined role of EG for cavity flow with association of non-Newtonian material. Das et al. ^19^ paid attention on EG framework involved due to microchannel. Ibrahim and Gamachu ^20^ explored the radiated onset of viscoelastic hybrid materials justifying by disk with optimized applications. Geridonmez and Oztop^21^ predicted exclusive features of entropy generation due to wavy conducting surface with magnetic particles. Wang et al.^22^ observed the Darcy Forchheimer analysis with EG effects numerically. Some further attempts on entropy generation impact is found in Refs.^23–27^.

Owing to the complex dynamic of non-Newtonian materials, the essential applications of such materials are noted in industrial and engineering mechanism. The wide importance of non-Newtonian materials is signified in polymers, manufacturing systems, food products, printing devices, oil pipelines etc. Various classes of non-Newtonian are observed due to complex rheological impact. The Casson fluid model is referred to this class with distinct consequences of shear thinning and thickening properties. The behavior of blood, ink, molten shows the nature of Casson model. Different investigations are presented dealing with this model. Khan et al.^28^ presented the numerical onset of Casson fluid model with oscillating unsteady regime. Bhatti et al.^29^ pronounced the Casson rheology with ion slip mechanism via differential transform method. Raza et al.^30^ reported a fractional computation for Casson model with inclined magnetic force impact. Haq et al.^31^ used bioconvective applications referring to Casson material with thermal transport phenomenon.

After observing a comprehensive literature survey on nanofluid and entropy generation phenomenon, the aim of current communication is to predict the assessment of heat and mass transfer aspects in flow of Casson nanofluid due to vertically moving plate. The novel objective of work is summarized as:

- Heat and mass transfer applications regarding the Casson nanofluid flow are reported with optimized framework.
- The fluid flow is subjecting to the chemical reaction and mixed convection consequences.
- The entropy generation and Bejan number significance is considered.
- The contribution of mixed convection and external heating source is discussed.
- The analytical simulations with Laplace technique are performed for set of flow parameters.
- The physical impact and applications of optimized phenomenon is evaluated graphically.

## Description of the problem

An unsteady laminar flow of Casson nanofluid is assumed due to vertical plate. The moving plate attains the variable velocity of $u\left( {0,\tau } \right) = A\tau$. For static plat, the fluid temperature and concentration is assumed to be $\theta_{\infty }$ and $C_{\infty }$, respectively. For moving plate, the fluid attains the uniform temperature $\theta_{w}$ and concentration $C_{w}$. The joule heating effects are ignored as it has been assumed that collision between fluid particles is small. The external heat source consequences are taken into account in heat equation. The modification in concentration equation is done with chemical reaction features. The governing equations under such assumptions are modeled as^23,24,29^:

1 $$ \rho \frac{\partial u(y,\tau )}{{\partial \tau }} = \mu \left( {1 + \frac{1}{\beta }} \right)\frac{{\partial^{2} u(y,\tau )}}{{\partial y^{2} }} + \rho g\beta_{\theta } (\theta (y,\tau ) - \theta_{\infty } ) + \rho g\beta_{c} (C(y,\tau ) - C_{\infty } ), $$

2 $$ \rho c_{p} \frac{\partial \theta (y,\tau )}{{\partial \tau }} = K\frac{{\partial^{2} \theta (y,\tau )}}{{\partial y^{2} }} + Q_{0} (\theta (y,\tau ) - \theta_{\infty } ), $$

3 $$ \frac{\partial C(y,\tau )}{{\partial \tau }} = D\frac{{\partial^{2} C(y,\tau )}}{{\partial y^{2} }} - K_{1} (C(y,\tau ) - C_{\infty } ) $$

These are associated with the following physical initial and boundary conditions^23^:

4 $$ \left. {\begin{array}{*{20}l} {u\left( {y,0} \right) = 0,} \hfill & {\theta \left( {y,0} \right) = \theta_{\infty } ,} \hfill & {{\text{C}}\left( {y,0} \right) = C_{\infty } } \hfill \\ {u\left( {0,\tau } \right) = A\tau ,} \hfill & {\theta \left( {0,\tau } \right) = \theta_{w} ,} \hfill & {{\text{C}}\left( {0,\tau } \right) = C_{w} } \hfill \\ {u\left( {\infty ,\tau } \right) = 0,} \hfill & {\theta \left( {\infty ,\tau } \right) = \theta_{\infty } ,} \hfill & {{\text{C}}\left( {\infty ,\tau } \right) = C_{\infty } } \hfill \\ \end{array} } \right\}, $$

The $\rho$ is the density, $u\left( {y,\tau } \right)$ is the velocity vector's x-component, $\beta$ is the Casson fluid's material parameter, $\mu$ is the dynamic viscosity, $g$ is the gravitational acceleration, $\beta_{\theta }$ is the volumetric thermal expansion, $\beta_{c}$ is the concentration coefficient, $\theta (y,\tau )$ is the temperature vector's x-component, $c_{p}$ is the heat capacitance, *k* is the fluid's thermal conductivity, $Q_{0}$ shows the heat generation, $k_{1}$ the reaction parameter and D for the mass diffusivity. Following non-dimensional variables are added to the Eqs. (1)–(4) in order to eliminate the units.

$$ u^{*} = \frac{u}{{\left( {vA} \right)^{\frac{1}{3}} }},\,{\text{ y}}^{*} = \frac{{yA^{\frac{1}{3}} }}{{\nu^{\frac{2}{3}} }},\, \, \tau^{*} = \frac{{\tau A^{\frac{2}{3}} }}{{\nu^{\frac{1}{3}} }},\,\,\,\,\theta^{*} (y,\tau ) = \frac{{\theta - \theta_{\infty } }}{{\theta_{w} - \theta_{\infty } }},{\text{ C}}^{*} (y,\tau ) = \frac{{C - C_{\infty } }}{{C_{w} - C_{\infty } }} $$

Into Eqs. (1)–(4),

5 $$ \frac{\partial u(y,\tau )}{{\partial \tau }} = \left( {1 + \frac{1}{\beta }} \right)\frac{{\partial^{2} u(y,\tau )}}{{\partial y^{2} }} + Gr\theta (y,\tau ) + GmC(y,\tau ), $$

6 $$ \Pr \frac{\partial \theta (y,\tau )}{{\partial \tau }} = \frac{{\partial^{2} \theta (y,\tau )}}{{\partial y^{2} }} + \delta \Pr \theta (y,\tau ), $$

7 $$ Sc\frac{\partial C(y,\tau )}{{\partial \tau }} = \frac{{\partial^{2} C(y,\tau )}}{{\partial y^{2} }} - \omega ScC(y,\tau ), $$

8 $$ \left. {\begin{array}{*{20}l} {u\left( {y,0} \right) = 0,} \hfill & {\theta \left( {y,0} \right) = 0,} \hfill & {{\text{C}}\left( {y,0} \right) = 0} \hfill \\ {u\left( {0,\tau } \right) = \tau ,} \hfill & {\theta \left( {0,\tau } \right) = 1,} \hfill & {{\text{C}}\left( {0,\tau } \right) = 1} \hfill \\ {u\left( {\infty ,\tau } \right) = 0,} \hfill & {\theta \left( {\infty ,\tau } \right) = 0,} \hfill & {{\text{C}}\left( {\infty ,\tau } \right) = 0} \hfill \\ \end{array} } \right\}, $$

where $Gr = \frac{{g\beta_{\theta } \Delta \theta }}{A}$ is the thermal Grashof number, $Gm = \frac{{g\beta_{c} \Delta C}}{A}$ is the mass Grashof number, ${\text{Pr}} = \frac{{\mu {\text{c}}_{{\text{p}}} }}{{\text{K}}}$ is the Prandtl number, ${\text{ Sc = }}\frac{\upsilon }{D}$ is the Schmidt number, $\delta = \frac{{Q_{0} }}{{\rho c_{p} }}A^{{ - \frac{2}{3}}} \upsilon^{\frac{1}{3}}$ is the heat generation parameter, $\omega = k_{1} A^{{ - \frac{2}{3}}} \upsilon^{\frac{1}{3}}$ is the chemical reaction parameter and $\upsilon$ fluid kinematic viscosity.

### Entropy generation

For the system described in Eqs. (5) to (8), the following giving entropy generation relations are created to maximize heat transmission and reduce energy losses^1,2,13^.

9 $$ E_{gen} = \frac{K}{{\theta_{\infty }^{2} }}\left( {\frac{\partial \theta }{{\partial y}}} \right)^{2} + \frac{D}{{C_{\infty }^{2} }}\left( {\frac{\partial C}{{\partial y}}} \right)^{2} + \frac{\mu }{{\theta_{\infty } }}\left( {1 + \frac{1}{\beta }} \right)\left( {\frac{\partial u}{{\partial y}}} \right)^{2} $$

A non-similarity variable is used to derive $\partial \theta /\partial y = \Delta \theta A^{\frac{1}{3}} \nu^{{ - \,\frac{2}{3}}} \partial \theta^{*} /\partial y^{*}$_,_ $\partial C/\partial y = \Delta CA^{\frac{1}{3}} \nu^{{ - \,\frac{2}{3}}} \partial C^{*} /\partial y^{*}$, and $\partial u/\partial y = A^{\frac{2}{3}} \nu^{{ - \,\frac{1}{3}}} \partial u^{*} /\partial y^{*}$ and added to Eq. (9), which results in

10 $$ N_{s} = \left[ {\left( {\frac{\partial \theta }{{\partial y}}} \right)^{2} + \frac{{D\zeta^{2} }}{{k\Omega^{2} }}\left( {\frac{\partial C}{{\partial y}}} \right)^{2} + \frac{{B_{r} }}{\Omega }\left( {1 + \frac{1}{\beta }} \right)\left( {\frac{\partial u}{{\partial y}}} \right)^{2} } \right] $$

where

$$ N_{s} = \frac{{E_{gen} \nu^{\frac{4}{3}} \theta^{2}_{\infty } }}{{kA^{2/3} (\Delta \theta )^{2} }},\, \, Br = \frac{{\mu A^{\frac{2}{3}} \nu^{\frac{2}{3}} }}{k\Delta \theta },\,\, \, \Omega = \frac{\Delta \theta }{{\theta_{\infty } }} = \frac{{\theta_{w} - \theta_{\infty } }}{{\theta_{\infty } }}, \, \zeta = \frac{\Delta C}{{C_{\infty } }} = \frac{{C_{w} - C_{\infty } }}{{C_{\infty } }} $$

### Bejan number

Bejan is credited with being the first author in the literature to identify a number of elements that can improve the efficiency of thermal system. He created Bejan number, which is the proportion of total entropy creation to the production of heat transfer entropy. Also proposed forth features of the second thermodynamic law that take into consideration a variety of problems with mixed convection. The source of the Bejan number is

11 $$ BN = \frac{{\frac{K}{{\theta_{\infty }^{2} }}\left( {\frac{\partial \theta }{{\partial y}}} \right)^{2} }}{{\frac{K}{{\theta_{\infty }^{2} }}\left( {\frac{\partial \theta }{{\partial y}}} \right)^{2} + \frac{D}{{C_{\infty }^{2} }}\left( {\frac{\partial C}{{\partial y}}} \right)^{2} + \frac{\mu }{{\theta_{\infty } }}\left( {1 + \frac{1}{\beta }} \right)\left( {\frac{\partial u}{{\partial y}}} \right)^{2} }} $$

and

12 $$ BN = \frac{{\left( {\frac{\partial \theta }{{\partial y}}} \right)^{2} }}{{\left( {\frac{\partial \theta }{{\partial y}}} \right)^{2} + \frac{{D\zeta^{2} }}{{k\Omega^{2} }}\left( {\frac{\partial C}{{\partial y}}} \right)^{2} + \frac{{B_{r} }}{\Omega }\left( {1 + \frac{1}{\beta }} \right)\left( {\frac{\partial u}{{\partial y}}} \right)^{2} }} $$

## Exact solutions by Laplace transform method

The governing problem is solved by using the Laplace transform technique. Recently, perturbation scheme is widely used in the different engineering and industrial problems, signal processing, control theory, plasma physics etc. The Laplace scheme can effectively tackle many complex problems in very simple way. The motivations for implementing this technique is due to fine accuracy. The perturbation technique does not involve any complicated discretization steps. The simulations are performed via MATHEMTICA software. Implementing the perturbation method on governing problem leads to:

13 $$ q\overline{u}(y,q) = \left( {1 + \frac{1}{\beta }} \right)\frac{{\partial^{2} \overline{u}(\eta ,q)}}{{\partial y^{2} }} + Gr\overline{\theta }(y,q) + Gm\overline{C}(y,q) $$

14 $$ \overline{u}\left( {0,q} \right) = \frac{1}{{q^{2} }},\,\,\,\,\,\,\overline{u}\left( {\infty ,q} \right) = 0 $$

15 $$ \Pr q\overline{\theta }\left( {y,q} \right) = \frac{{\partial^{2} \overline{\theta }(y,q)}}{{\partial y^{2} }} + \delta \Pr \overline{\theta }\left( {y,q} \right) $$

16 $$ \overline{\theta }\left( {0,q} \right) = \frac{1}{q},\,\,\,\,\,\,\overline{\theta }\left( {\infty ,q} \right) = 0 $$

17 $$ Sc \, q\overline{C}\left( {y,q} \right) = \frac{{\partial^{2} \overline{C}(y,q)}}{{\partial y^{2} }} - Sc \, \omega \overline{C}\left( {y,q} \right) $$

18 $$ \overline{C}\left( {0,q} \right) = \frac{1}{q},\,\,\,\,\,\,\overline{C}\left( {\infty ,q} \right) = 0 $$

The transformation boundary conditions (16) are used to solve the second order differential Eq. (15).

19 $$ \overline{\theta }\left( {y,q} \right) = \frac{{e^{{ - y\sqrt {(q - \delta )\Pr } }} }}{q} $$

The Laplace transformation is inverted to produce

20 $$ \theta \left( {y,\tau } \right) = \frac{1}{2}\left[ {e^{{ - y\sqrt { - \delta \Pr } }} erfc\left( {\frac{{y\sqrt {\Pr } }}{2\sqrt \tau } - \sqrt { - \delta \Pr \tau } } \right) + e^{{y\sqrt { - \delta \Pr } }} erfc\left( {\frac{{y\sqrt {\Pr } }}{2\sqrt \tau } + \sqrt { - \delta \Pr \tau } } \right)} \right] $$

The transformation boundary conditions (18) are used to solve the second order differential Eq. (17).

21 $$ \overline{C} \left( {y,q} \right) = \frac{{e^{{ - y\sqrt {(q + \omega )Sc} }} }}{q} $$

Inverting the Laplace transform yields

22 $$ C\left( {y,\tau } \right) = \frac{1}{2}\left[ {e^{{ - y\sqrt {\omega Sc} }} erfc\left( {\frac{{y\sqrt {Sc} }}{2\sqrt \tau } - \sqrt {\omega Sc\tau } } \right) + e^{{y\sqrt {\omega Sc} }} erfc\left( {\frac{{y\sqrt {Sc} }}{2\sqrt \tau } + \sqrt {\omega Sc\tau } } \right)} \right] $$

Similar to how the answer of Eq. (13) becomes

23 $$ \begin{aligned} \overline{u}(y,q) = & \frac{1}{{q^{{^{2} }} }}e^{{ - y\sqrt {\lambda q} }} - \frac{1}{{a_{0} }}\frac{1}{q}e^{{ - y\sqrt {\lambda q} }} + \frac{1}{{b_{0} }}\frac{1}{q}e^{{ - y\sqrt {\lambda q} }} + \frac{1}{{a_{0} }}\frac{1}{q - A}e^{{ - y\sqrt {\lambda q} }} \\ & - \frac{1}{{b_{0} }}\frac{1}{q + B}e^{{ - y\sqrt {\lambda q} }} - \frac{1}{{a_{0} }}\frac{1}{q - A}e^{{ - y\sqrt {\Pr } \sqrt {q - \delta } }} + \frac{1}{{a_{0} }}\frac{1}{q}e^{{ - y\sqrt {\Pr } \sqrt {q - \delta } }} \\ & + \frac{1}{{b_{0} }}\frac{1}{q + B}e^{{ - y\sqrt {Sc} \sqrt {q + \omega } }} - \frac{1}{{b_{0} }}\frac{1}{q}e^{{ - y\sqrt {Sc} \sqrt {q + \omega } }} \\ \end{aligned} $$

as

$$a_{0} = \frac{{\delta {\text{ Pr}}}}{\lambda Gr}, \, a_{1} = \frac{{{\text{ Pr}} - \lambda }}{\lambda Gr}{, }b_{0} = \frac{{\omega {\text{ Sc}}}}{\lambda Gm}{\text{, b}}_{1} = \frac{{{\text{ Sc}} - \lambda }}{\lambda Gm}{\text{, A = }}\frac{{a_{0} }}{{a_{1} }}{\text{, B = }}\frac{{b_{0} }}{{b_{1} }}{, }\frac{1}{\lambda }{ = 1 + }\frac{1}{\beta }$$.

Applying inverse Laplace transformation,

24 $$ u(y,\tau ) = u_{1} (y,\tau ) + y_{2} (y,\tau ) + u_{3} (y,\tau ) + u_{4} (y,\tau ) $$

Such as the

25 $$ u_{1} (y,\tau ) = \left( {\tau + \frac{{\lambda y^{2} }}{2}} \right)erfc\left( {\frac{\sqrt \lambda y}{{2\sqrt \tau }}} \right) - \sqrt \lambda y\sqrt {\frac{\tau }{\pi }} e^{{ - \frac{{\lambda y^{2} }}{4\tau }}} - \frac{1}{{a_{0} }}erfc\left( {\frac{\sqrt \lambda y}{{2\sqrt \tau }}} \right) + \frac{1}{{b_{0} }}erfc\left( {\frac{\sqrt \lambda y}{{2\sqrt \tau }}} \right) $$

26 $$ \begin{aligned} u_{2} (y,\tau ) = & \frac{1}{{2a_{0} }}e^{A\tau } \left[ {e^{{ - y\sqrt {\lambda A} }} erfc\left( {\frac{\sqrt \lambda y}{{2\sqrt \tau }} - \sqrt {A\tau } } \right) + e^{{y\sqrt {\lambda A} }} erfc\left( {\frac{\sqrt \lambda y}{{2\sqrt \tau }} + \sqrt {A\tau } } \right)} \right] \\ & - \frac{1}{{2b_{0} }}e^{ - B\tau } \left[ {e^{{ - y\sqrt { - \lambda B} }} erfc\left( {\frac{\sqrt \lambda y}{{2\sqrt \tau }} - \sqrt { - B\tau } } \right) + e^{{y\sqrt { - B\lambda } }} erfc\left( {\frac{\sqrt \lambda y}{{2\sqrt \tau }} + \sqrt { - B\tau } } \right)} \right] \\ \end{aligned} $$

27 $$ \begin{aligned} u_{3} (y,\tau ) = & - \frac{1}{{2a_{0} }}e^{A\tau } \left( {e^{{ - y\sqrt {\Pr (A - \delta )} }} erfc \, \left( {\frac{{y\sqrt {\Pr } }}{2\sqrt \tau } - \sqrt {(A - \delta )\tau } } \right) + e^{{y\sqrt {\Pr (A - \delta )} }} erfc \, \left( {\frac{{y\sqrt {\Pr } }}{2\sqrt \tau } + \sqrt {(A - \delta )\tau } } \right)} \right) \\ & { + }\frac{1}{{2a_{0} }}\left( {e^{{ - y\sqrt { - \delta \Pr } }} erfc \, \left( {\frac{{y\sqrt {\Pr } }}{2\sqrt \tau } - \sqrt { - \delta \tau } } \right) + e^{{y\sqrt { - \delta \Pr } }} erfc \, \left( {\frac{{y\sqrt {\Pr } }}{2\sqrt \tau } + \sqrt { - \delta \tau } } \right)} \right) \\ \end{aligned} $$

28 $$ \begin{aligned} u_{4} (y,\tau ) = & \frac{1}{{2b_{0} }}e^{ - B\tau } \left( {e^{{ - y\sqrt {Sc(\omega - B)} }} erfc \, \left( {\frac{{y\sqrt {Sc} }}{2\sqrt \tau } - \sqrt {(\omega - B)\tau } } \right) + e^{{y\sqrt {Sc(\omega - B)} }} erfc \, \left( {\frac{{y\sqrt {Sc} }}{2\sqrt \tau } + \sqrt {(\omega - B)\tau } } \right)} \right) \\ & - \frac{1}{{2b_{0} }}\left( {e^{{ - y\sqrt {\omega Sc} }} erfc \, \left( {\frac{{y\sqrt {Sc} }}{2\sqrt \tau } - \sqrt {\omega \tau } } \right) + e^{{y\sqrt {\omega Sc} }} erfc \, \left( {\frac{{y\sqrt {Sc} }}{2\sqrt \tau } + \sqrt {\omega \tau } } \right)} \right) \\ \end{aligned} $$

### Skin friction

The non-dimensional form of skin friction defined as

29 $$ c_{f} = \left( {1 + \frac{1}{\beta }} \right)\left. {\frac{\partial v(y,\tau )}{{\partial y}}} \right|_{y = 0} $$

Table: variations in several variables for ${C}_{f}$.

### Nusselt number

The non-dimensional version of the heat transfer rate is given as,

30 $$ Nu = \left. {\frac{\partial \theta (y,\tau )}{{\partial y}}} \right|_{y = 0} $$

## Results and discussion

**Figure 1:**
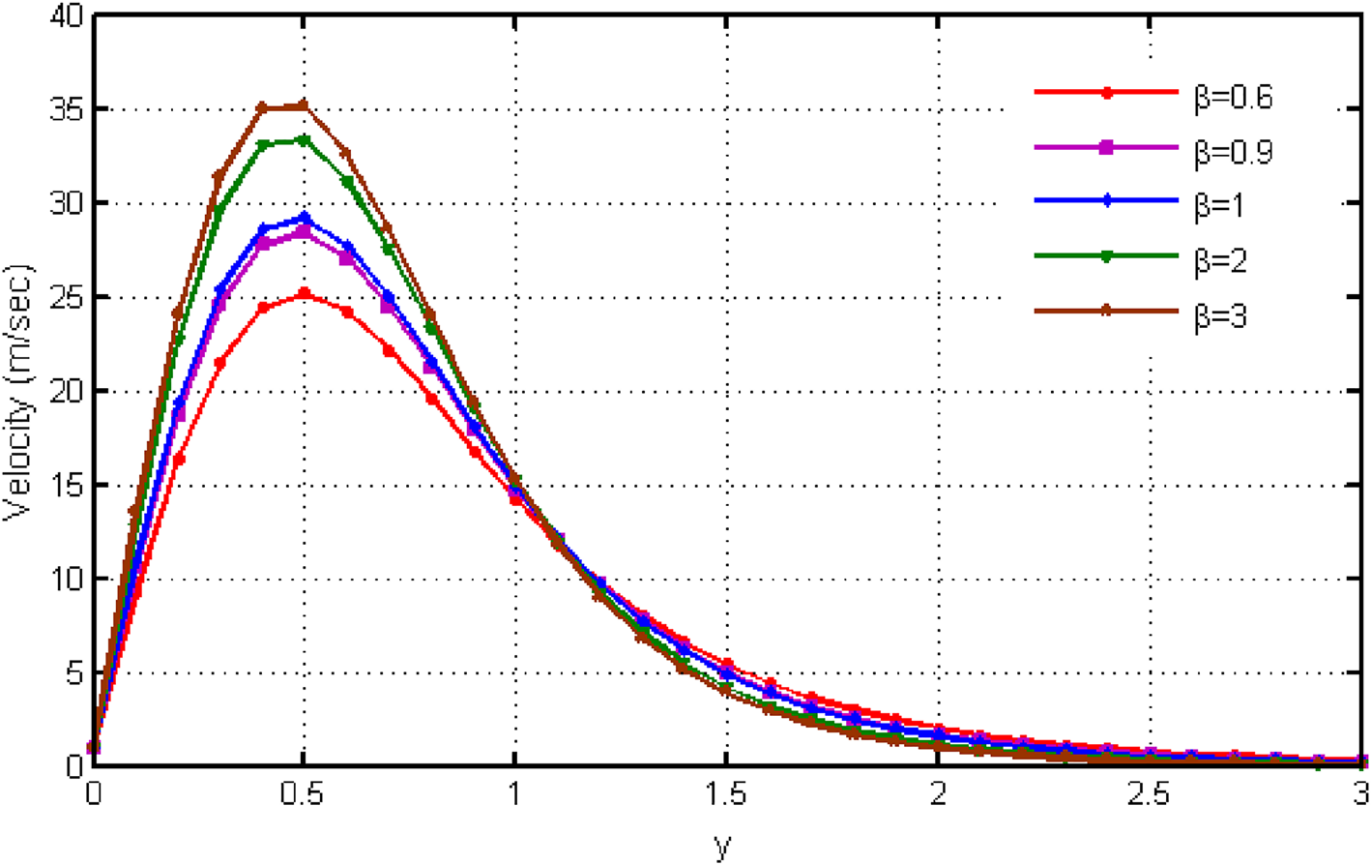
Variation in velocity profile for different values of C_6_H_9_NaO_7_ parameter $\beta$.

**Figure 2:**
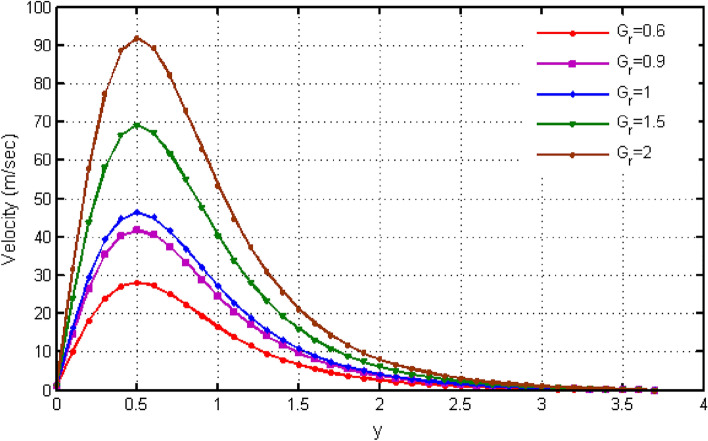
Variation in velocity profile for different values of $Gr$.

**Figure 3:**
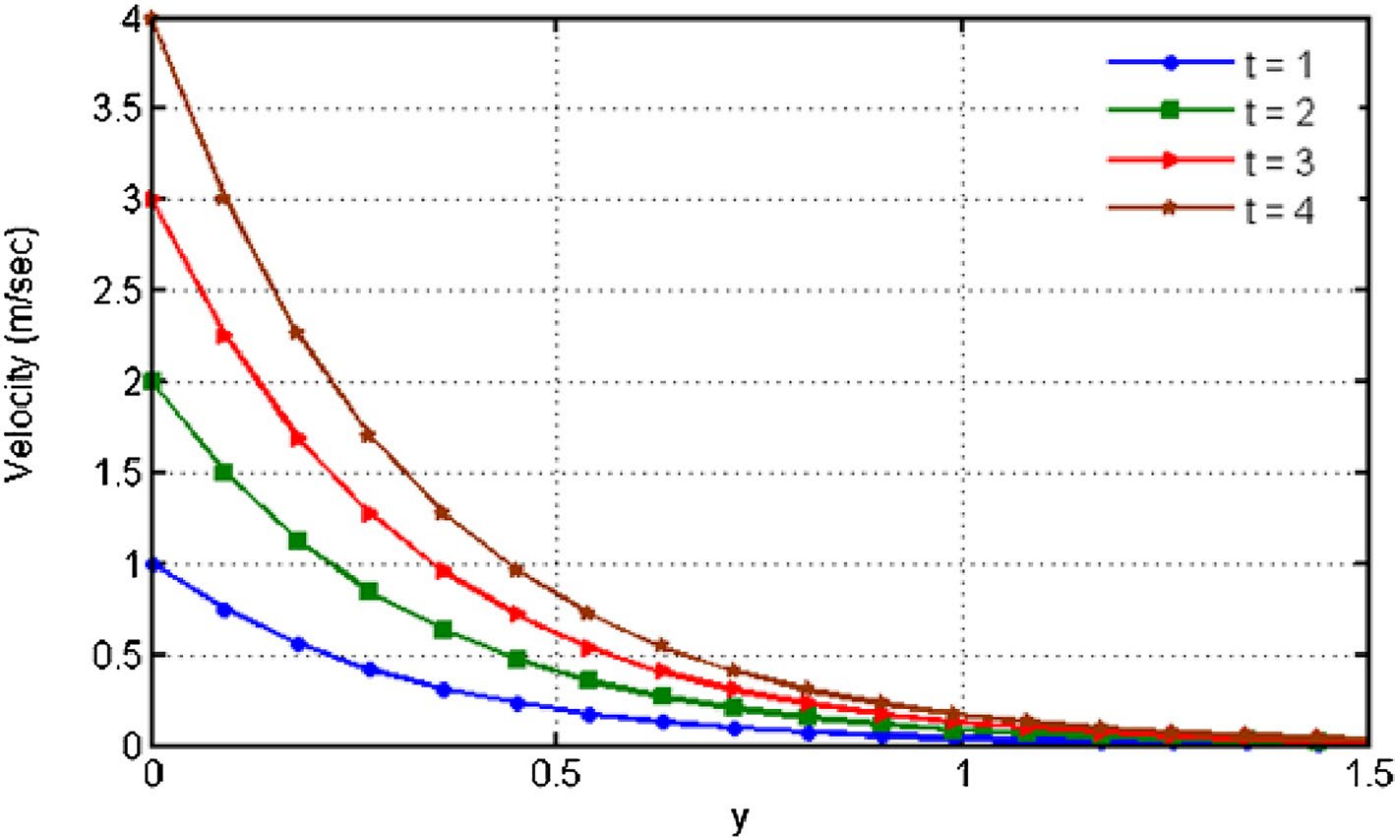
Variation in velocity profile for different values of time $t$.

**Figure 4:**
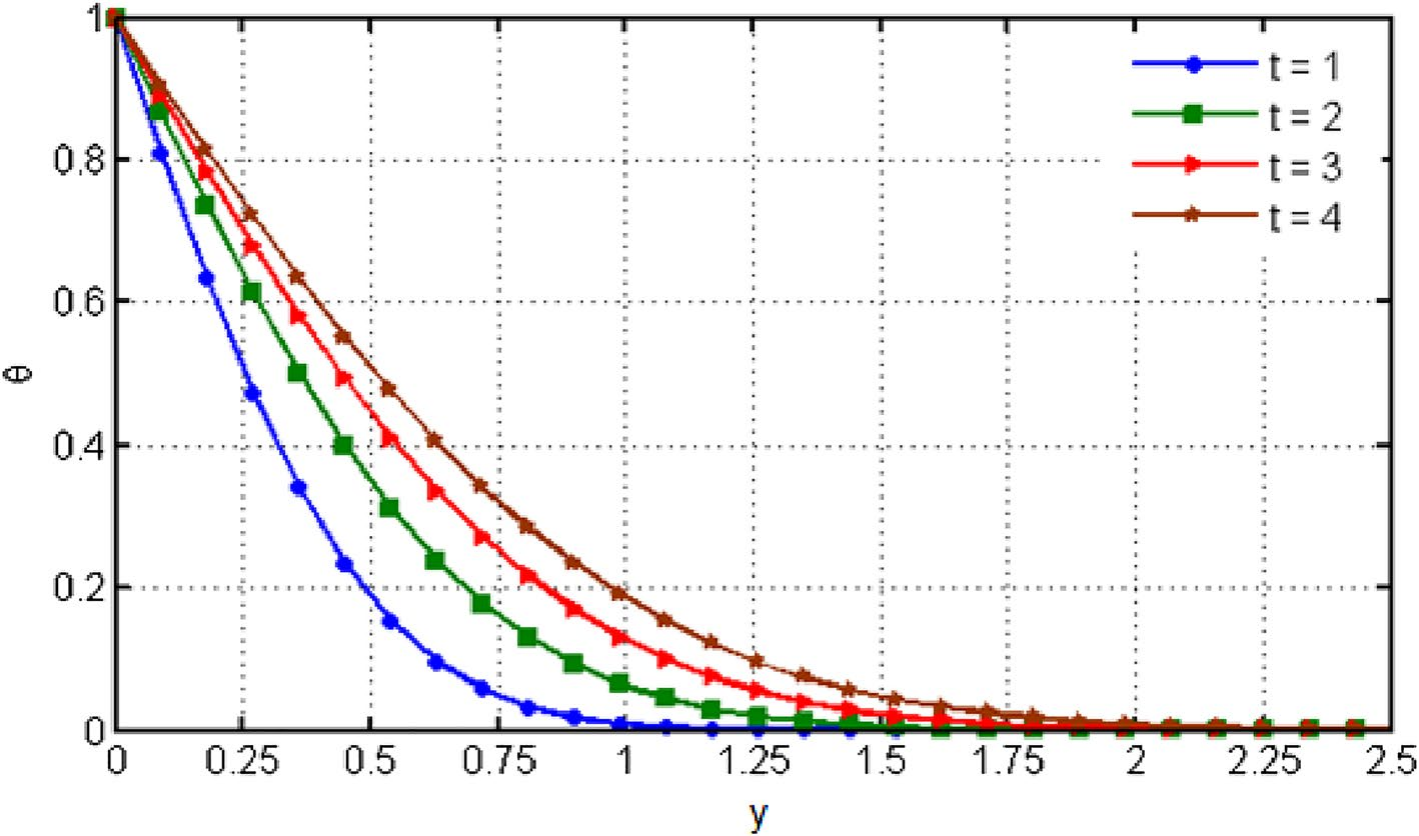
Variation in temperature profile for different values of time $t$.

**Figure 5:**
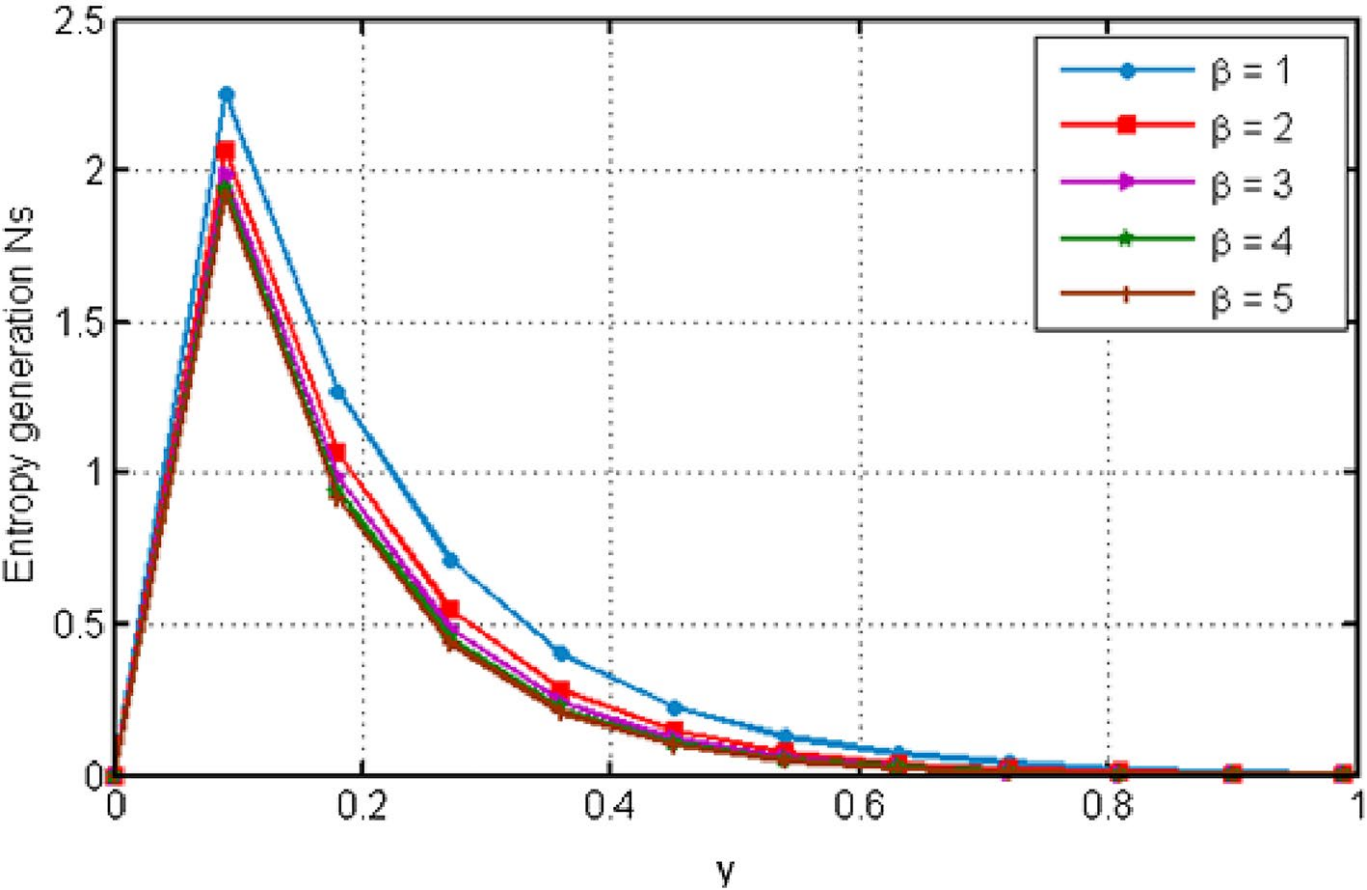
Variation in entropy generation for different values of C_6_H_9_NaO_7_ parameter $\beta$.

**Figure 6:**
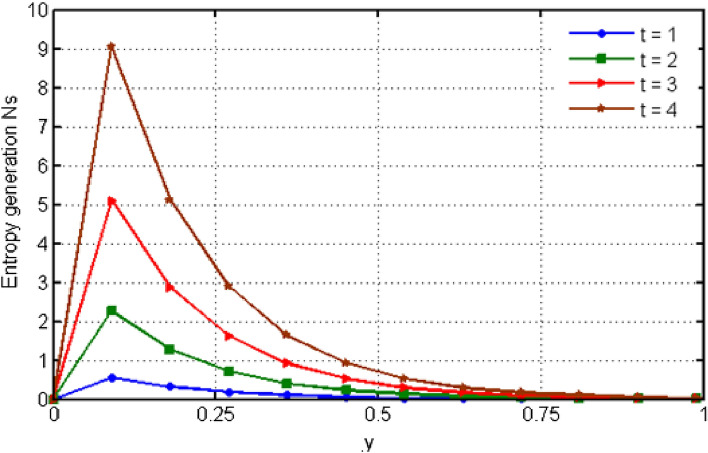
Variation in entropy generation for different values of time $t$.

**Figure 7:**
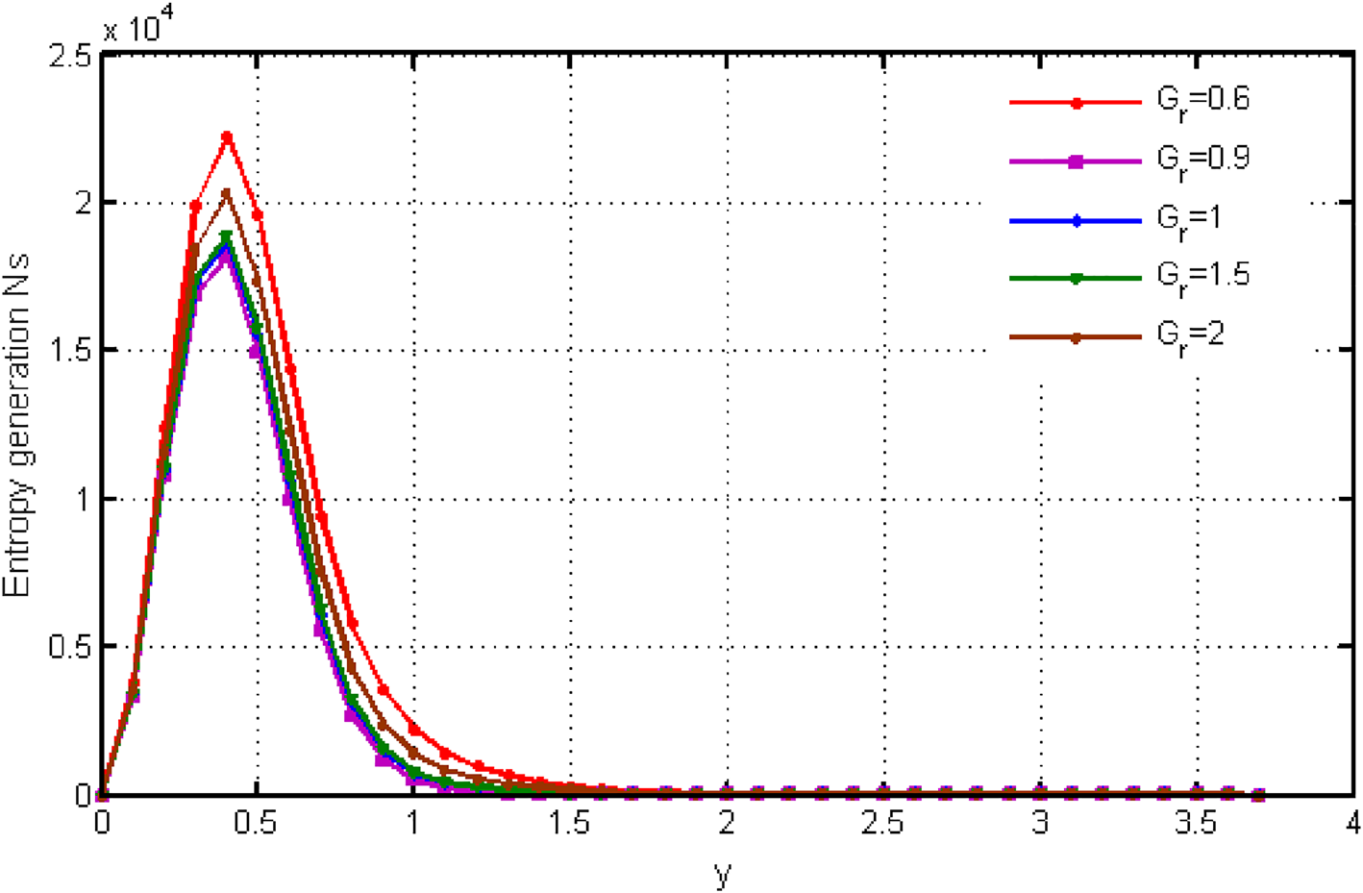
Variation in entropy generation for different values of $Gr$.

**Figure 8:**
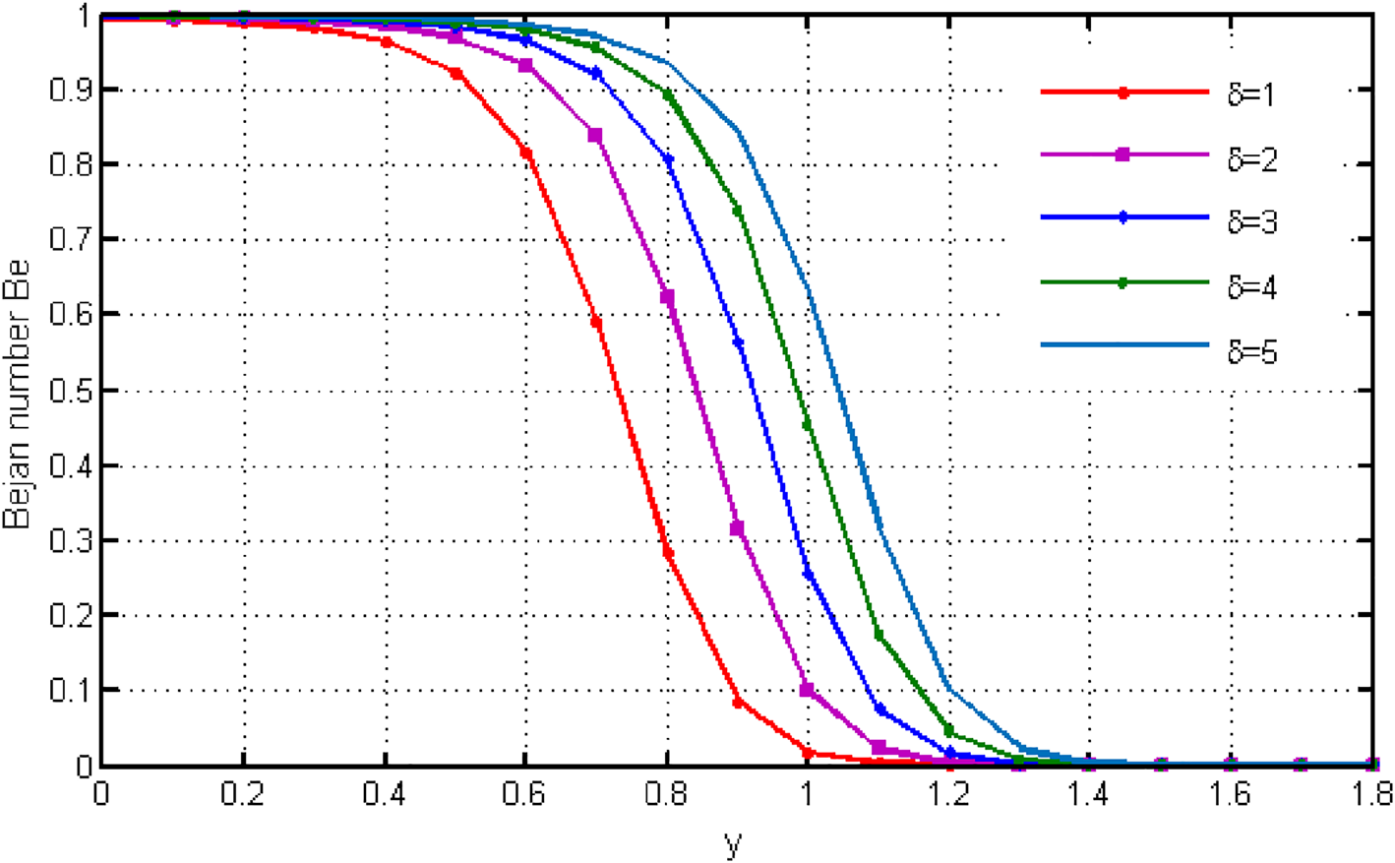
Variation in Bejan number for different values of heat generation parameter $\delta$.

**Figure 9:**
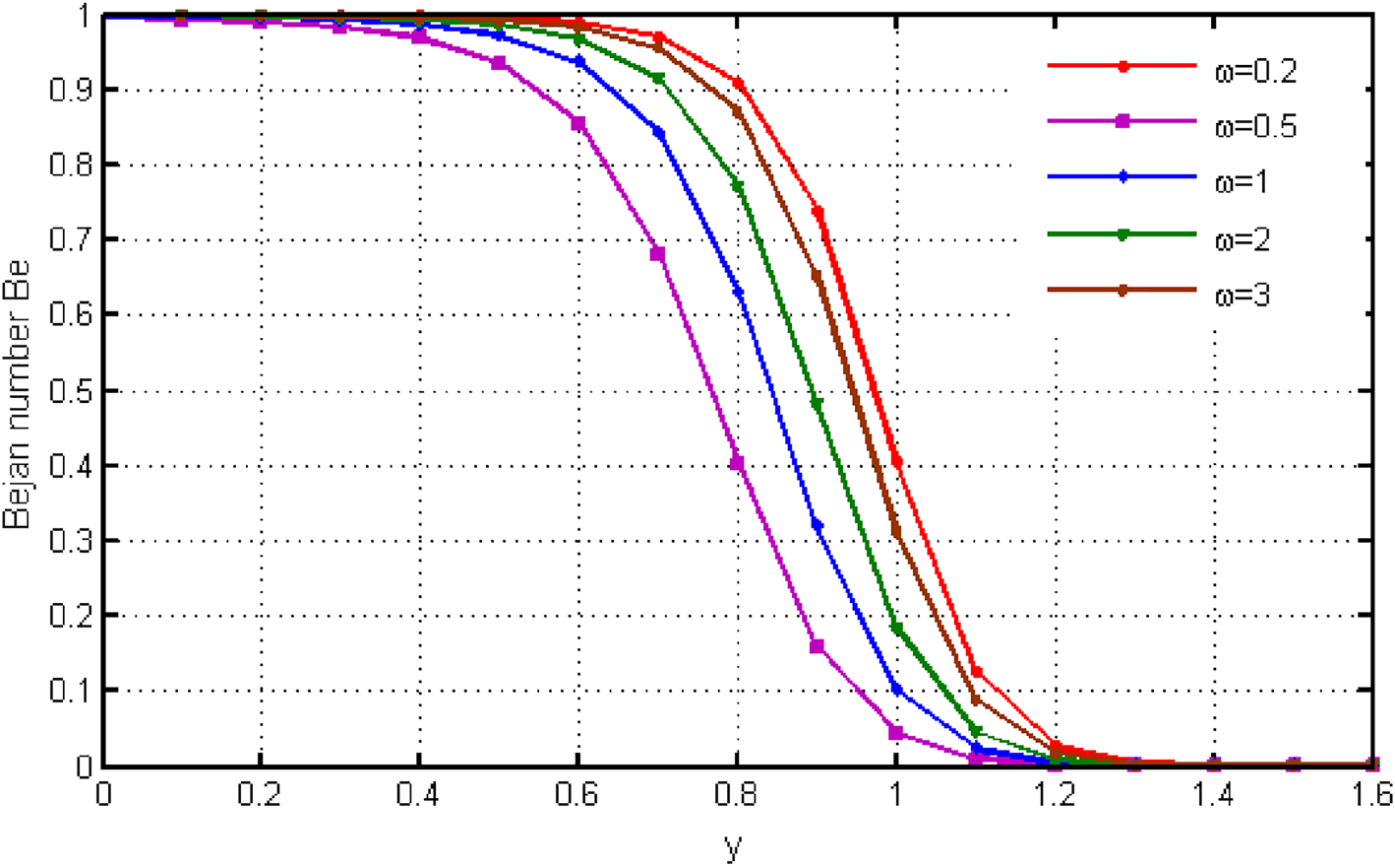
Variation in Bejan number for different values of chemical reaction parameter $\omega$.

The study of EG (entropy generation) for the flow of an accelerating non-Newtonian fluid was examined in this paper. The 2nd law of thermodynamics commonly known as EG which is highly helpful in solving heating contribution as analyzing the heat ex-changer. This section demonstrate the effect of many factors that influence the fluid flow. Furthermore, the graphical analysis is used in this section to show the velocity, entropy generation, temperature, and Bejan number. Current model is based on theoretical flow assumptions. Each parameter is assigned some fixed numerical value like $Gr = 0.5,$, $Gm = 0.4,$ ${\text{ Sc = }}0.2,$, $\delta = 0.3,$, $\omega = 0.2.$ The value of Prandtl number is taken as 13.09. The value of $\Pr = 13.09$ is obtained by combining the values of $\mu = 0.002;\,k = 0.6376\,\,{\text{and}}\,\,c_{p} = 4175$ into the fixed value of Pr which can be computed using the formula $\Pr = \mu c_{p} /k.$ Fig. 1 depicts the effect of $\beta$ (Casson-parameter) and it can be seen that the dual effect is produced. The velocity of the fluid initially exhibits increasing behaviour close to the plate, but as go away from the plate, this behaviour reverse and exhibits decreasing effects for greater values of $\beta$. Physically, upon enlarging $\beta$, the thickness of the boundary layer decreases which causes a drop in velocity. The variation in the velocity profile for various $Gr$ values is shown in Fig. 2. It is evident from the figure that the increasing values of $Gr$ rises the value of velocity. It is clear that velocity increases with increasing value of $Gr$, and this is because $Gr$ increases the buoyancy force, which increases velocity of the fluid. Grashof number illustrates the impact of temperature on fluid velocities and the proportion of buoyancy to viscous force. The variations in velocity profile for various values of time $t$ is shown in Fig. 3. Since unstable flow has been considered, the graph demonstrates this pattern by showing how the fluid velocity increases as time $t$ goes on. The liquid goes thought to be time-dependent, and as a result, velocity rises over time $t$. The impact of time $t$ on the temperature profile is illustrated in Fig. 4. The temperature of the fluid rises as time $t$ increases. The fluid's temperature reaches its maximum over extended periods of time. The fluctuation in entropy generation Ns for various Casson-parameter $\beta$ values is shown in Fig. 5. The graph shows that for greater values of $\beta$, the entropy generation Ns decreases. Physically, such consequences are referred to the shear thinning and thickening consequences of Casson fluid model. The fluctuation in entropy generation for various values of the time is seen in Fig. 6. The figure shows that the generation of entropy grows as time goes on. Figure 7 shows how the formation of entropy varies for various values of the $Gr$. The amount of entropy generated decreases for large values of $Gr.$ Figure 8 referring the thermal association of Bejan number due to enhancing character of heating generation factor. The improvement in Bejan assessment is predicted for larger heat generation parameter. In Fig. 9, the prediction for Bejan number due to chemical reaction parameter is noted. A decreasing aspect of Bejan number is observed with this parameter.

**Figure 10:**
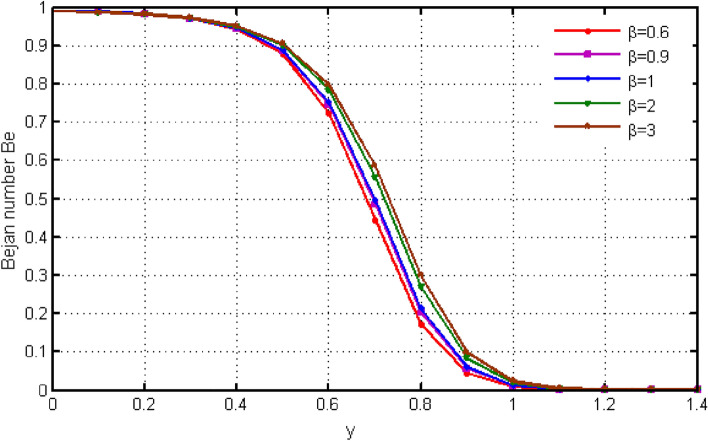
Variation in Bejan number for different values of C_6_H_9_NaO_7_ parameter $\beta$.

**Figure 11:**
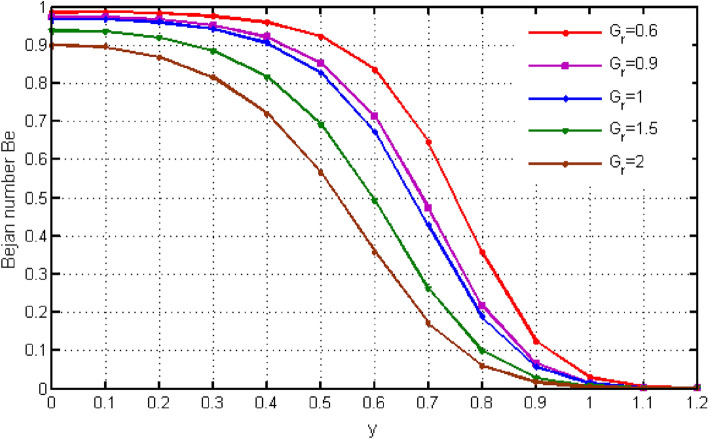
Variation in Bejan number for different values of $Gr$.

**Figure 12:**
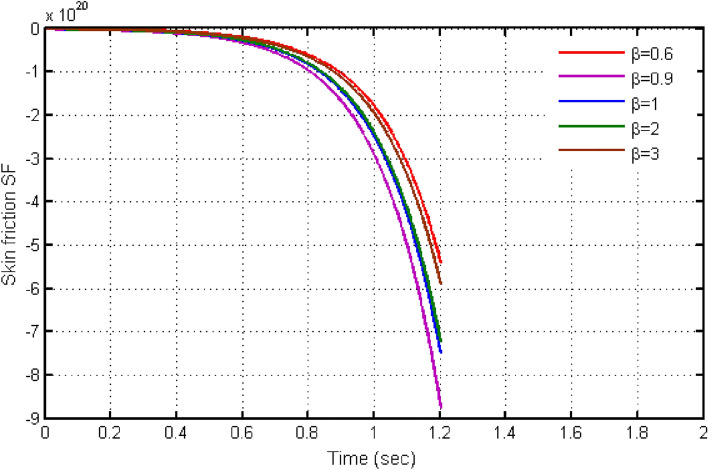
Variation in skin-friction for different values of C_6_H_9_NaO_7_ parameter $\beta$.

**Figure 13:**
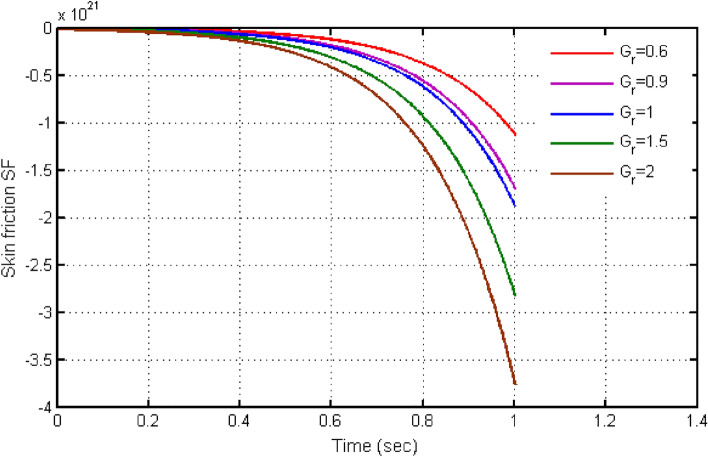
Variation in skin-friction for different values of $Gr$.

From Fig. 10, it can be seen that raising the values of $\beta$ causes the Bejan number to rise. Initially Be shows small variations. Instant after, Be exhibits larger variation for higher values of $\beta$. The significant determination of $Gr$ defining Bejan applications is continued in Fig. 11. Leading to $Gr$ values cause the Bejan number to fall. Figures 12 and 13 demonstrate the findings for skin friction. The effects of skin friction are deduced via Fig. 12 for b. It is discovered that skin friction reduces with higher values of $\beta$. This behaviour is completely the opposite to the velocity and is consistent with the physical environment. According to Fig. 13, the fluctuation in skin friction with rising Grashof number is not very obvious, but if one looks closely, the skin friction rises with increased $Gr$. Such effects is due to presence of buoyancy force.

Table 1 displays the results of our finite difference approach. The numerical information in the table illustrates the variance in fluid entropy generation velocity. These findings demonstrate that EG velocity decreases with rising values $\beta$. Table 2 executed that decreasing assessment of velocity is pronounced for improving $Gr.$ Table 3 confirmed that larger velocity fluctuation is noted for porosity factor. Table 4 concentrates that velocity magnitude is enhancing for impact of $Pr.$ In Tables 5, 6 and 7, larger variation is observed for $Sc$, $\delta$ and $\omega .$

**Table 1:** Numerical framework regarding velocity $\beta$.

β	t	Pr	Gr	y	U	BN	Ns	SF
1	2	14	0.05	0.5	0.73923	0.133032	0.00223	− 0.68272
2	2	14	0.05	0.5	0.71742	0.19844	0.00125	− 0.55234
3	2	14	0.05	0.5	0.70862	0.233432	0.00125	− 0.47454
4	2	14	0.05	0.5	0.70543	0.254534	0.00123	− 0.43283
5	2	14	0.05	0.5	0.69268	0.27493	0.00123	− 0.39202

**Table 2:** Numerical framework regarding velocity Gr.

Gr	β	t	Pr	y	u	Ns	SF
0.5	1	2	14	0.5	1.5148	0.0205	− 1.4419
1	1	2	14	0.5	2.3764	0.0640	− 2.2873
2	1	2	14	0.5	4.0998	0.2339	− 3.9782
3	1	2	14	0.5	5.8231	0.4811	− 5.6691
4	1	2	14	0.5	7.5464	0.8356	− 7.3600

**Table3:** Numerical framework regarding velocity $Gm$.

Gm	β	t	pr	Gr	y	u	BN	Ns	SF
5	1	2	14	0.05	0.5	0.7720	0.1260	0.0022	− 0.7126
10	1	2	14	0.05	0.5	0.8538	0.1108	0.0025	− 0.7917
15	1	2	14	0.05	0.5	0.9356	0.0980	0.0029	− 0.8707
20	1	2	14	0.05	0.5	1.0174	0.0873	0.0032	− 0.9498
25	1	2	14	0.05	0.5	1.0992	0.0782	0.0036	− 1.0289

**Table 4:** Numerical framework regarding velocity for $Pr.$

Pr	β	t	Gr	y	u	Theta	Nu	BN	Ns	SF
10	1	2	0.05	0.5	0.7440	0.1606	0.0116	0.0821	0.0016	− 0.6931
14	1	2	0.05	0.5	0.8058	0.1710	0.0168	0.1147	0.0025	− 0.7493
20	1	2	0.05	0.5	1.4599	0.2579	0.0344	0.0688	0.0172	− 1.2429
25	1	2	0.05	0.5	5.7994	0.4637	0.0742	0.0135	0.4065	− 4.0317
30	1	2	0.05	0.5	38.2756	1.0018	0.1851	0.0019	18.1901	− 22.1790

**Table 5:** Numerical framework regarding velocity for $\delta .$

$\delta$	β	t	pr	Gr	y	u	BN	Ns	SF
− 0.8	1	2	14	0.05	0.5	0.7077	0.1330	0.0012	− 0.6517
− 0.5	1	2	14	0.05	0.5	0.7078	0.1979	0.0012	− 0.6517
0	1	2	14	0.05	0.5	0.7086	0.2335	0.0012	− 0.6526
0.5	1	2	14	0.05	0.5	0.7937	0.2556	0.0024	− 0.7361
0.8	1	2	14	0.05	0.5	3.4008	0.2706	1.3338	− 3.0696

**Table 6:** Numerical framework regarding velocity for $Sc$.

Sc	β	t	Pr	Gr	y	u	BN	C	Ns	SF
10	1	2	14	0.05	0.5	0.8058	0.1147	0.0014	0.0025	− 0.7493
15	1	2	14	0.05	0.5	0.7706	0.1237	$1.3772 \times 10^{ - 4}$	0.0023	− 0.7100
20	1	2	14	0.05	0.5	0.7515	0.1286	$1.8187 \times 10^{ - 5}$	0.0022	− 0.6906
25	1	2	14	0.05	0.5	0.7396	0.1316	$3.0051 \times 10^{ - 6}$	0.0021	− 0.6790
30	1	2	14	0.05	0.5	0.7315	0.1337	$5.9843 \times 10^{ - 7}$	0.0021	− 0.6713

**Table 7:** Numerical framework regarding velocity for $\omega .$

$\omega$	β	t	Pr	Gr	y	u	BN	C	Ns	SF
0.05	1	2	14	0.05	0.5	0.8280	0.1073	0.0020	0.0026	− 0.7739
0.3	1	2	14	0.05	0.5	0.8150	0.1116	0.0016	0.0025	− 0.7595
0.5	1	2	14	0.05	0.5	0.8058	0.1147	0.0014	0.0025	− 0.7493
0.8	1	2	14	0.05	0.5	0.7937	0.1188	0.0011	0.0024	− 0.7361
1	1	2	14	0.05	0.5	0.7867	0.1212	$9.0040 \times 10^{ - 4}$	0.0023	− 0.7285

## Conclusions

Entropy generation applications for Casson nanofluid against the vertical plate have been observed. The analysis is supported with chemical reaction, mixed convection and external heating source impact. The Laplace technique is utilized on the governing model. The graphical assessment for parameters is discussed. The numerical calculation of problem is presented by varying different flow parameters. Major findings are:

- With increasing Grashof number and Casson fluid parameter, the velocity profile enhanced.
- The enhancement in heat transfer is predicted at larger time instant.
- A control of entropy generation is noticed for larger Casson fluid parameter.
- An enhancing impact of Grashof number is noted for optimized phenomenon.
- Progressive change in heat generation parameter improves the Bejan number.
- Declining results for Bejan number due to larger reaction parameter has been observed.
- The reducing magnitude of wall shear force is exhibited for Casson fluid parameter.
- The obtained results present applications in thermal devices, energy solar systems, heat transfer enhancement, mechanical systems, manufacturing processes etc.
- The modification in current model can be further updated for hybrid nanofluid, sensitivity analysis, nonlinear radiation phenomenon, activation energy, bioconvection etc.

## Data Availability

The datasets used and/or analyzed during the current study available from the corresponding author on reasonable request.

## List of symbols

*u*: Velocity of the fluid, (ms^−^^1^)
*θ*: Temperature of the fluid, (K)
C: Concentration of the fluid
*g*: Acceleration due to gravity, (ms^−^^2^)
*c*_*p*_: Specific heat at a constant pressure, (J Kg^−^^1^ K^−^^1^)
*Gr*: Thermal Grasshof number
*k*: Thermal conductivity of the fluid, (W m^−^^2^ K^−^^1^)
Gm: The mass Grashof number
Sc: The Schmidt number
*ω*: The chemical reaction parameter
*Nu*: Nusselt number
Pr: Prandtl number, (μ*c*_*p*_*/k*)
*θ*: Fluid temperature far away from the plate, (K)
*q*: Laplace transforms parameter
*K*: Porosity parameter
D: The mass diffusivity
*k*^1^: The chemical reaction parameter
*Q*_0_: The heat generation term
*δ*: The heat generation parameter
A: Random constant (ms^−2^)

## Greek symbols

*ν*: Kinematic viscosity of the fluid, (m^2^s^−^^1^)
*μ*: Dynamic viscosity, (kg m^−^^1^ s^−^^1^)
*ρ*: Fluid density, (kg ms^−^^3^)
*β*_0_: The volumetric coefficient of thermal expansion, (*K*^−^^1^)
*β*: The Casson fluid parameter
*β*_c_: The coefficient of concentration
*Br*: The Brinkman number
Ω: The dimensionless temperature function
